# Supplementary material for: Personalised analytics for rare disease diagnostics
Source: Nat Commun. 2019 Nov 21;10:5274. doi: 10.1038/s41467-019-13345-5 (PMC6872807; doi:10.1038/s41467-019-13345-5)
Supplement: Supplementary file 1 — Supplementary Information [file 41467_2019_13345_MOESM1_ESM.pdf]

Supplementary Information

Personalised analytics for rare disease diagnostics

Anderson et al.

Performance of VARPP compared to CADD or MetaSVM scores alone when using GTEx expression

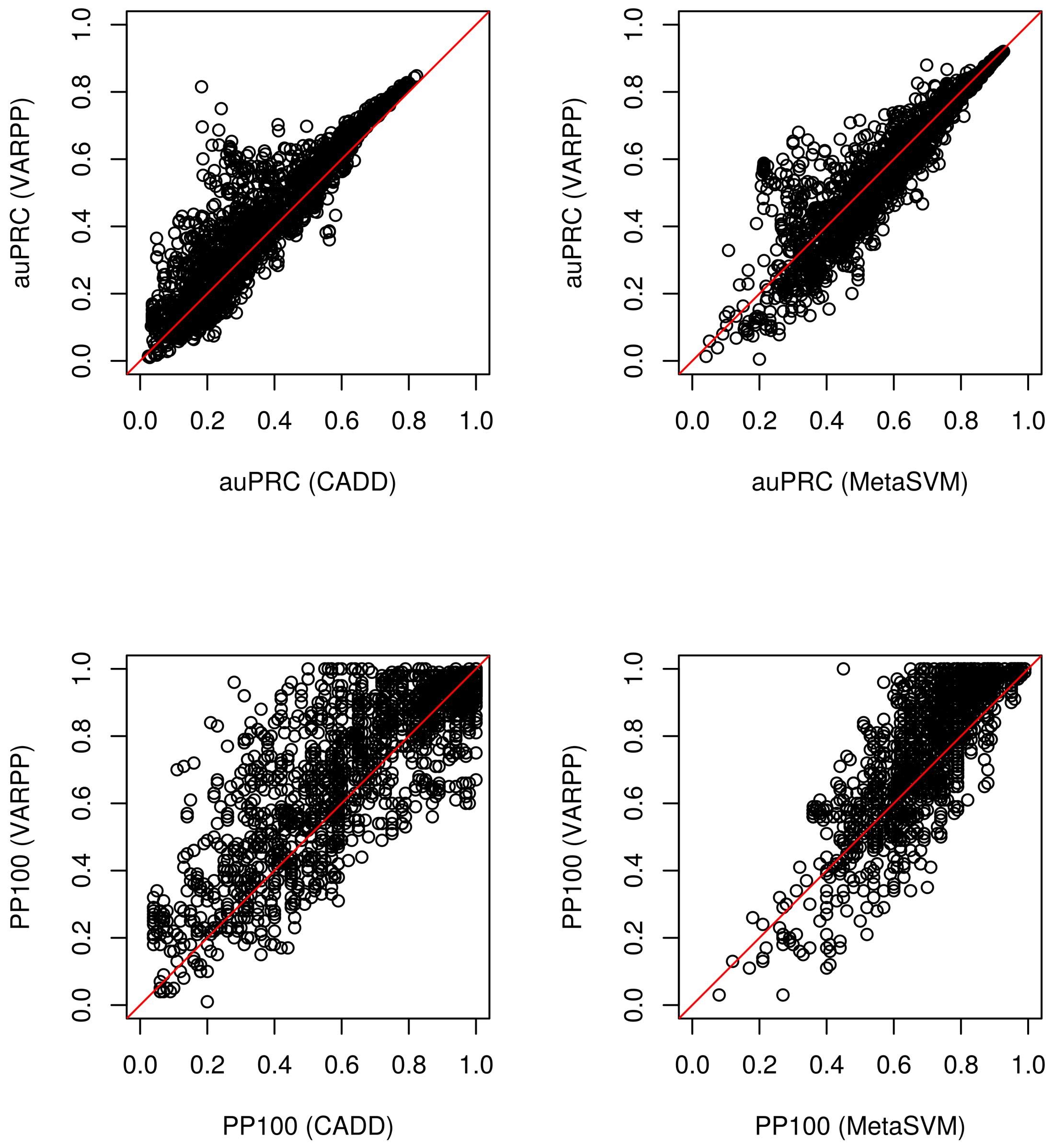

Supplementary Figure 1: Top left Agreement scatter plot comparing the auPRC for VARPP including CADD + GTEx expression (*y* axis) versus the auPRC for CADD scores alone (*x* axis). **Top right** Agreement scatter plot comparing the auPRC for VARPP including MetaSVM + GTEx expression (*y* axis) versus the auPRC for MetaSVM scores alone (*x* axis). **Bottom left** Agreement scatter plot comparing the PP100 for VARPP including CADD + GTEx expression (*y* axis) versus the auPRC for CADD scores alone (*x* axis). **Bottom right** Agreement scatter plot comparing the PP100 for VARPP including MetaSVM + GTEx expression (*y* axis) versus the auPRC for MetaSVM scores alone (*x* axis). The red line is the line of identity.

Performance of VARPP compared to CADD or MetaSVM scores alone when using GTEx specificity

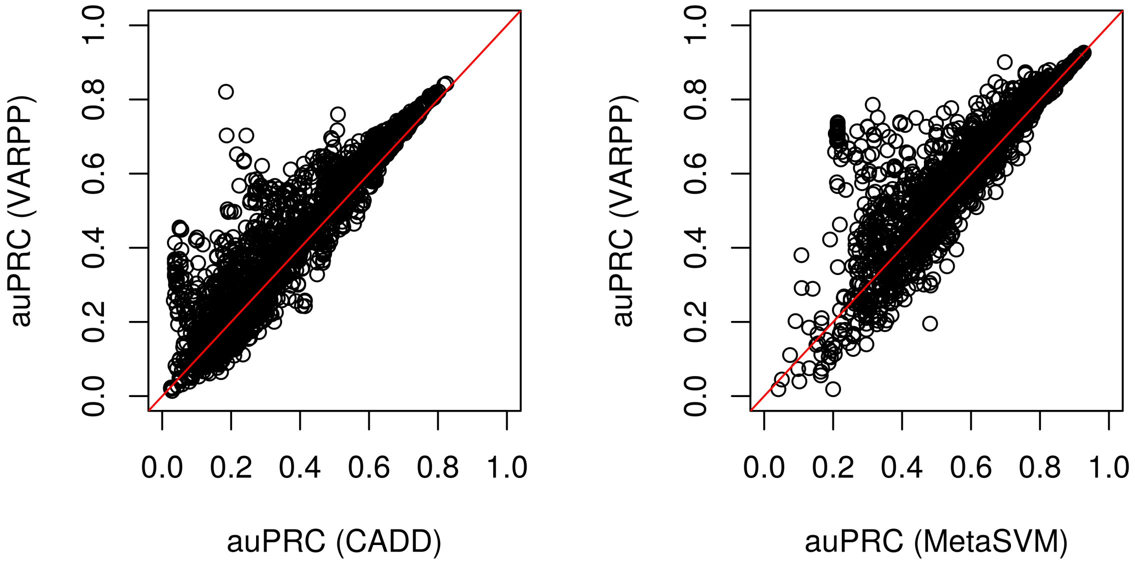

Supplementary Figure 2: Top left Agreement scatter plot comparing the auPRC for VARPP including CADD + GTEx specificity (*y* axis) versus the auPRC for CADD scores alone (*x* axis). **Top right** Agreement scatter plot comparing the auPRC for VARPP including MetaSVM + GTEx specificity (*y* axis) versus the auPRC for MetaSVM scores alone (*x* axis). The red line is the line of identity.

Performance of VARPP compared to CADD or MetaSVM scores alone when using FANTOM5 expression

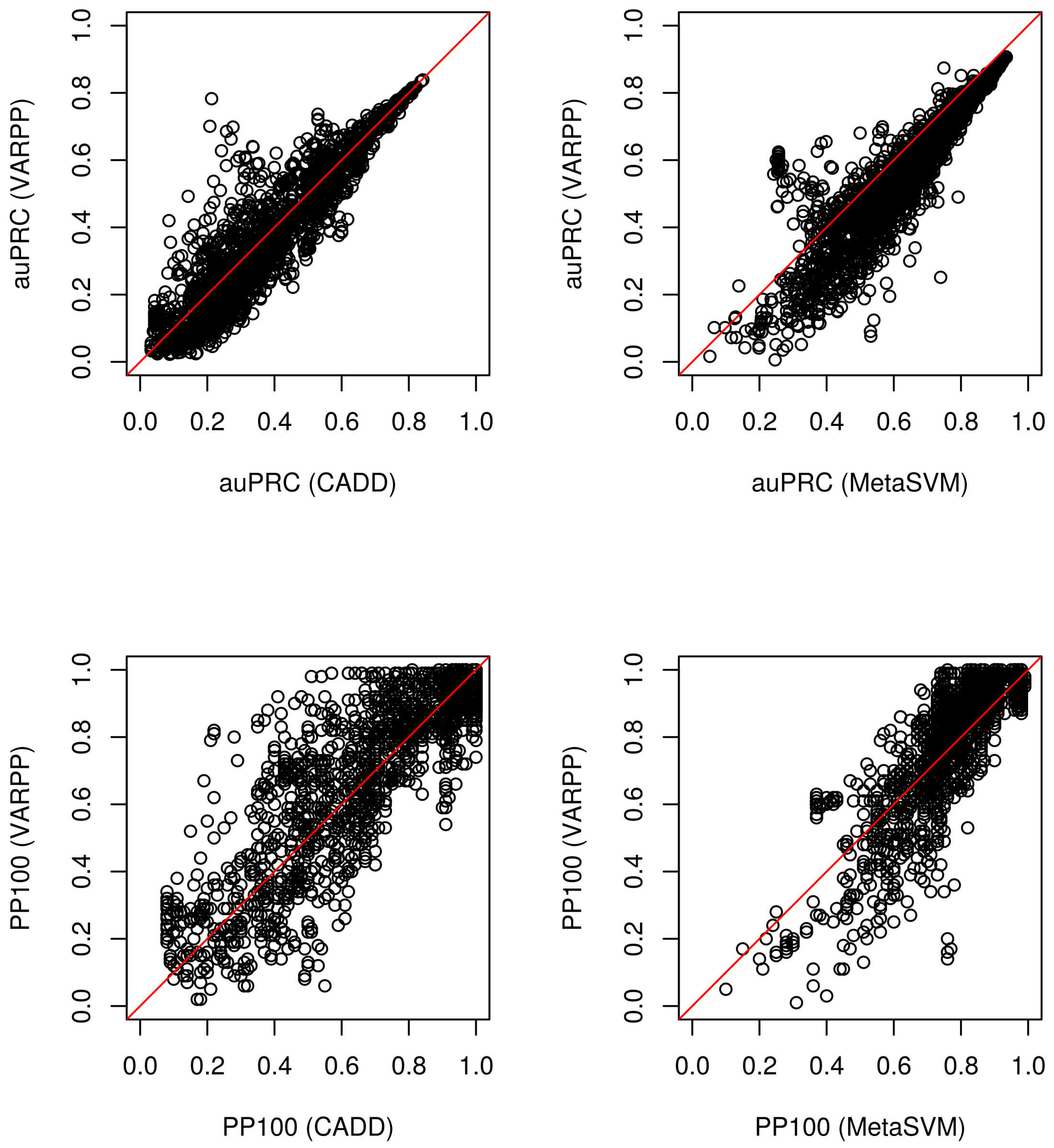

Supplementary Figure 3: **Top left** Agreement scatter plot comparing the auPRC for VARPP including CADD + FANTOM5 expression (*y* axis) versus the auPRC for CADD scores alone (*x* axis). **Top right** Agreement scatter plot comparing the auPRC for VARPP including MetaSVM + FANTOM5 expression (*y* axis) versus the auPRC for MetaSVM scores alone (*x* axis). **Bottom left** Agreement scatter plot comparing the PP100 for VARPP including CADD + FANTOM5 expression (*y* axis) versus the auPRC for CADD scores alone (*x* axis). **Bottom right** Agreement scatter plot comparing the PP100 for VARPP including MetaSVM + FANTOM5 expression (*y* axis) versus the auPRC for MetaSVM scores alone (*x* axis). The red line is the line of identity.

Performance of VARPP compared to CADD or MetaSVM scores alone when using FANTOM5 specificity

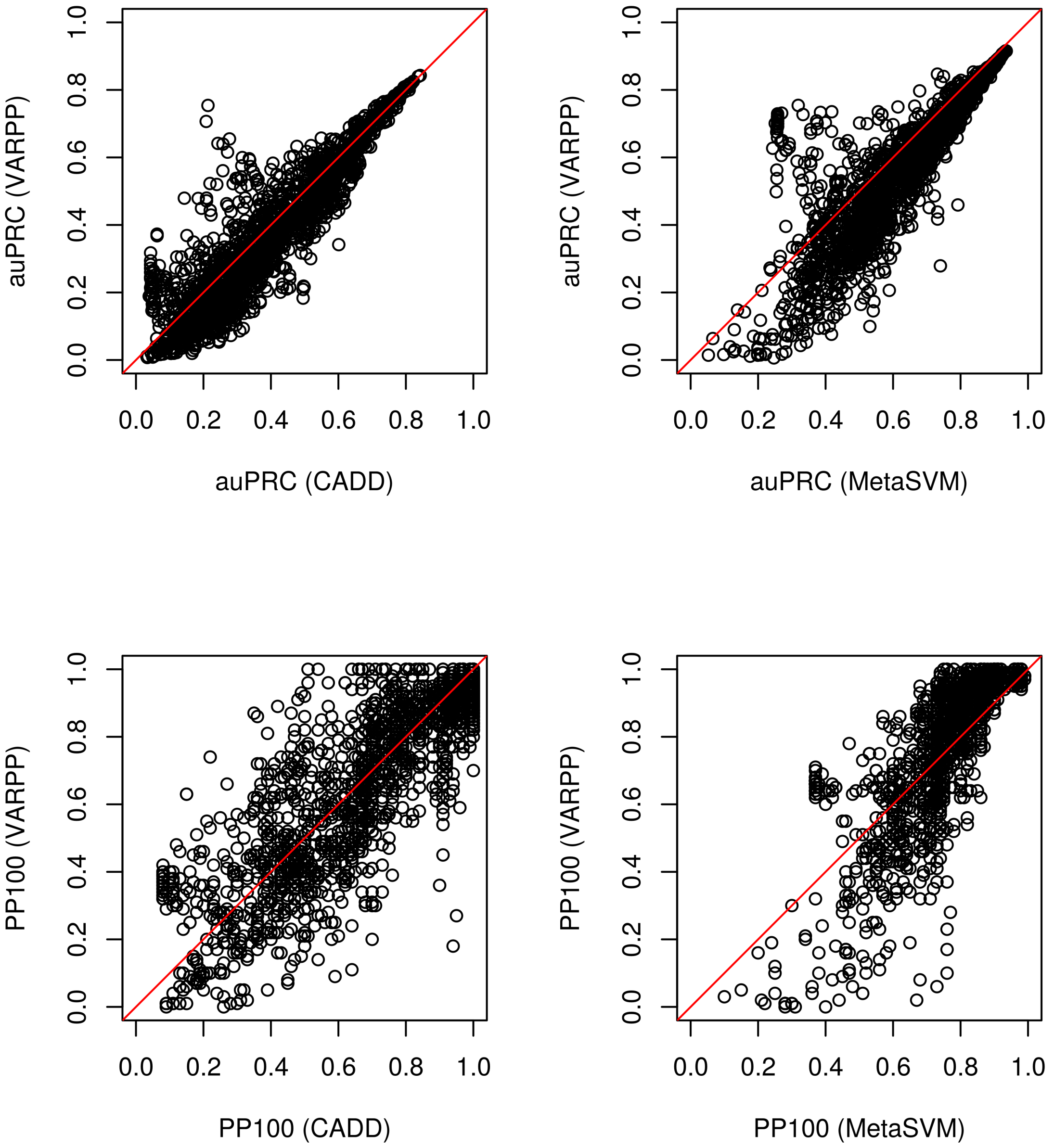

Supplementary Figure 4: Top left Agreement scatter plot comparing the auPRC for VARPP including CADD + FANTOM5 specificity (*y* axis) versus the auPRC for CADD scores alone (*x* axis). **Top right** Agreement scatter plot comparing the auPRC for VARPP including MetaSVM + FANTOM5 specificity (*y* axis) versus the auPRC for MetaSVM scores alone (*x* axis). **Bottom left** Agreement scatter plot comparing the PP100 for VARPP including CADD + FANTOM5 specificity (*y* axis) versus the auPRC for CADD scores alone (*x* axis). **Bottom right** Agreement scatter plot comparing the PP100 for VARPP including MetaSVM + FANTOM5 specificity (*y* axis) versus the auPRC for MetaSVM scores alone (*x* axis). The red line is the line of identity.

Performance of VARPP compared to CADD scores alone by disease group

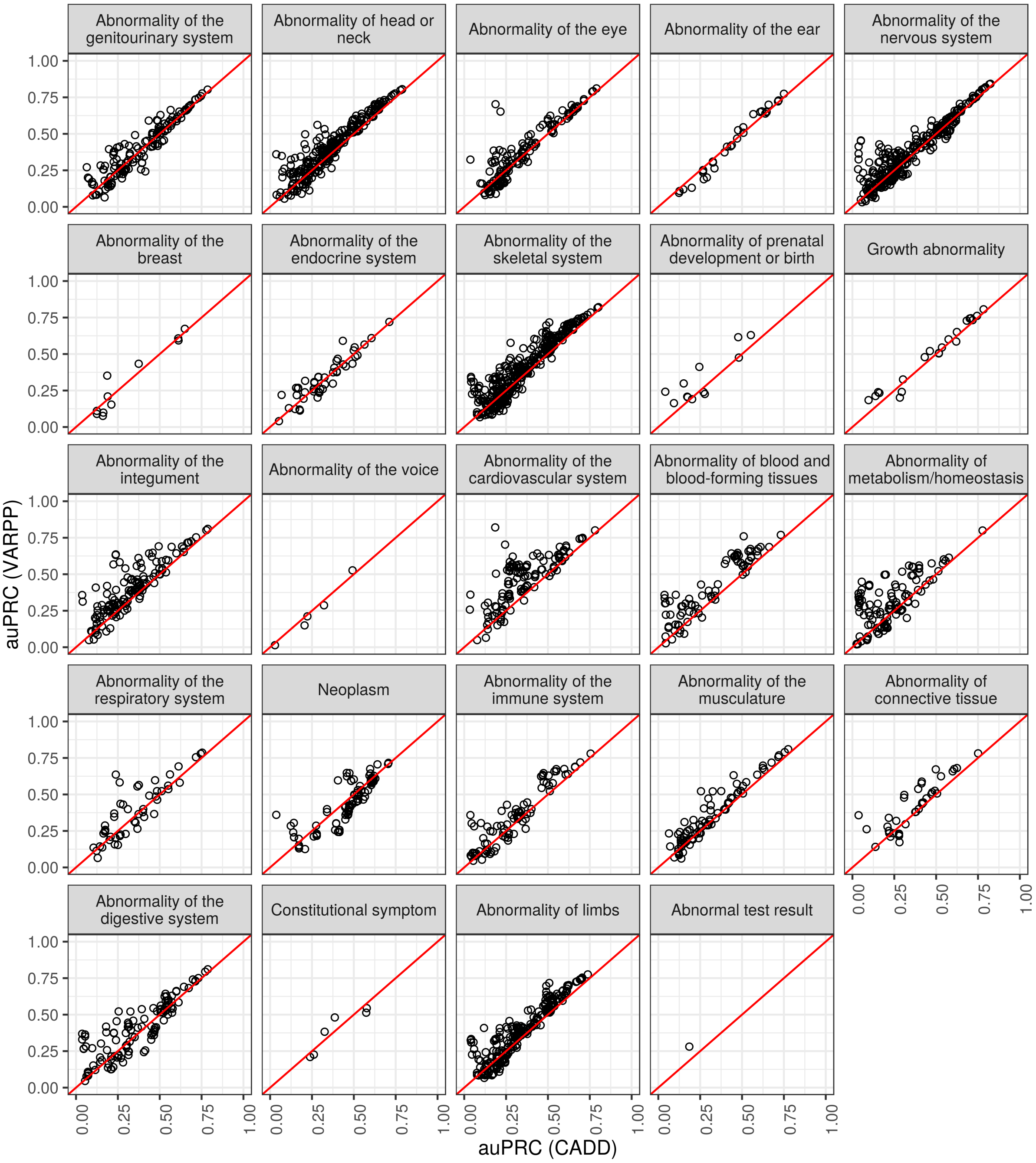

Supplementary Figure 5: Agreement scatter plots comparing the auPRC for VARPP including CADD + GTEx specificity (*y* axis) versus the auPRC for CADD scores alone (*x* axis) across broad HPO disease groups. The red line is the line of identity.

Performance of VARPP compared to MetaSVM scores alone by disease group

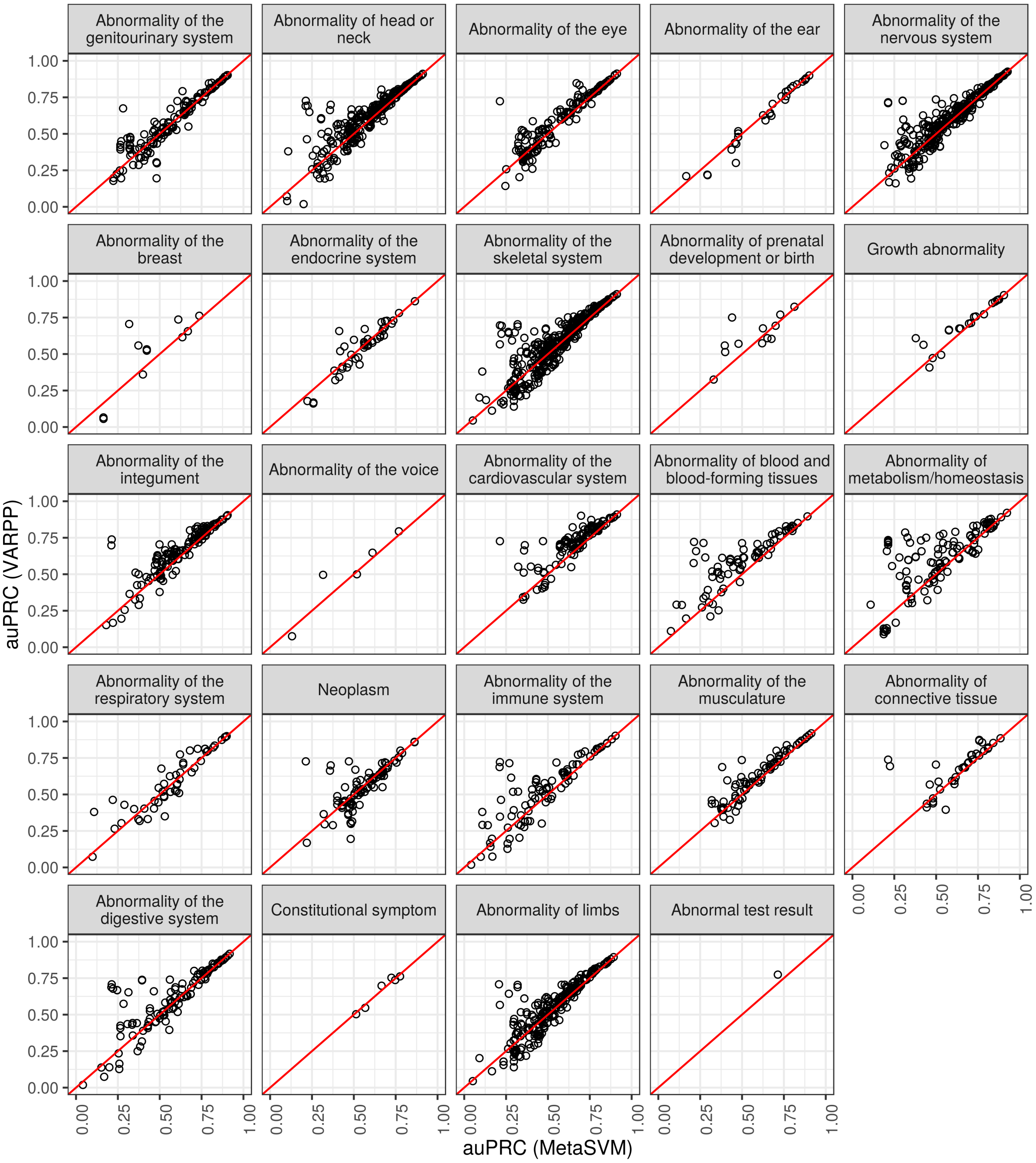

Supplementary Figure 6: Agreement scatter plots comparing the auPRC for VARPP including MetaSVM + GTEx specificity (*y* axis) versus the auPRC for MetaSVM scores alone (*x* axis) across broad HPO disease groups. The red line is the line of identity.

Performance of VARPP compared to CADD scores alone by disease group

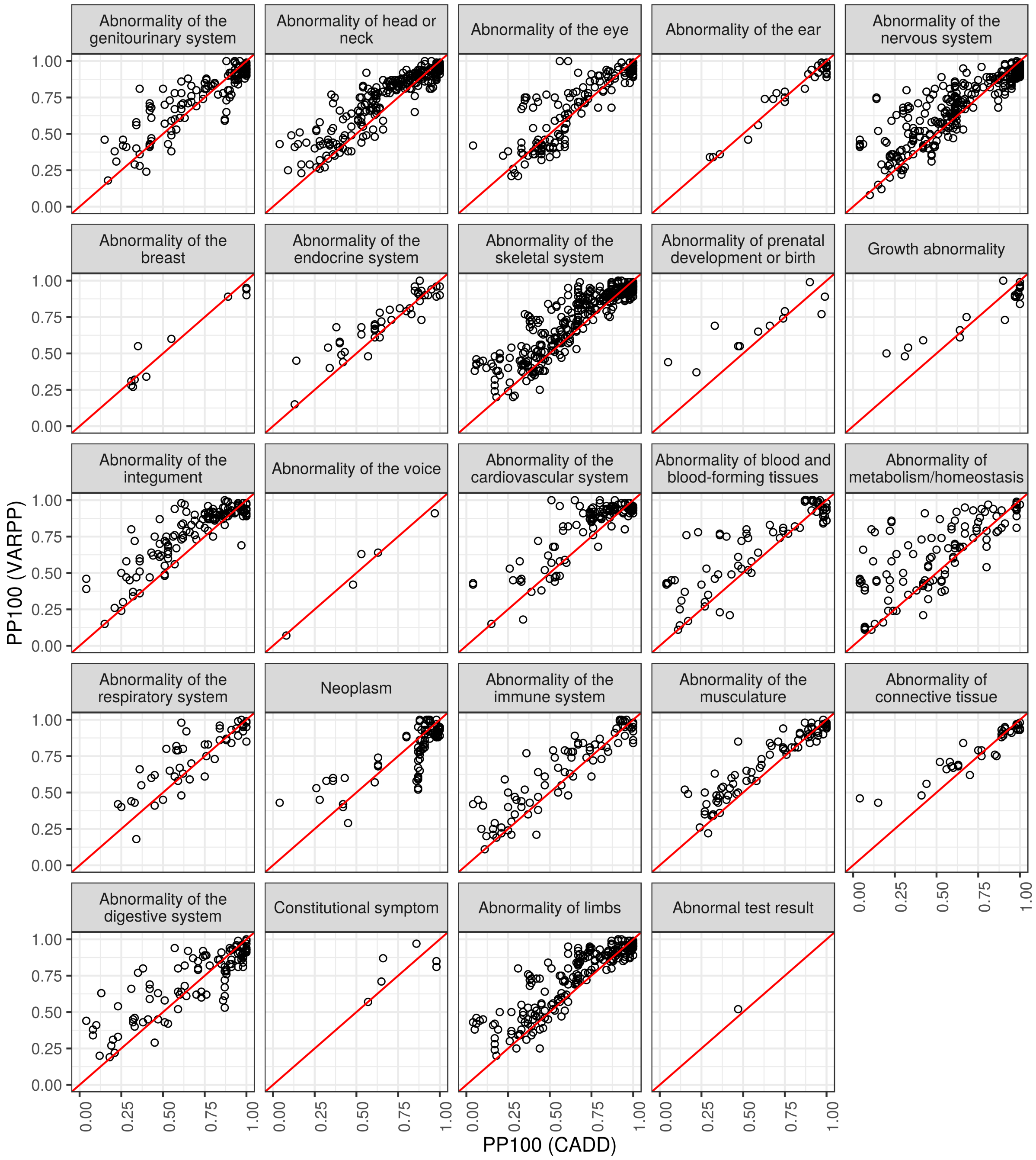

Supplementary Figure 7: Agreement scatter plots comparing the PP100 for VARPP including CADD + GTEx specificity (*y* axis) versus the PP100 for CADD scores alone (*x* axis) across broad HPO disease groups. The red line is the line of identity.

Performance of VARPP compared to MetaSVM scores alone by disease group

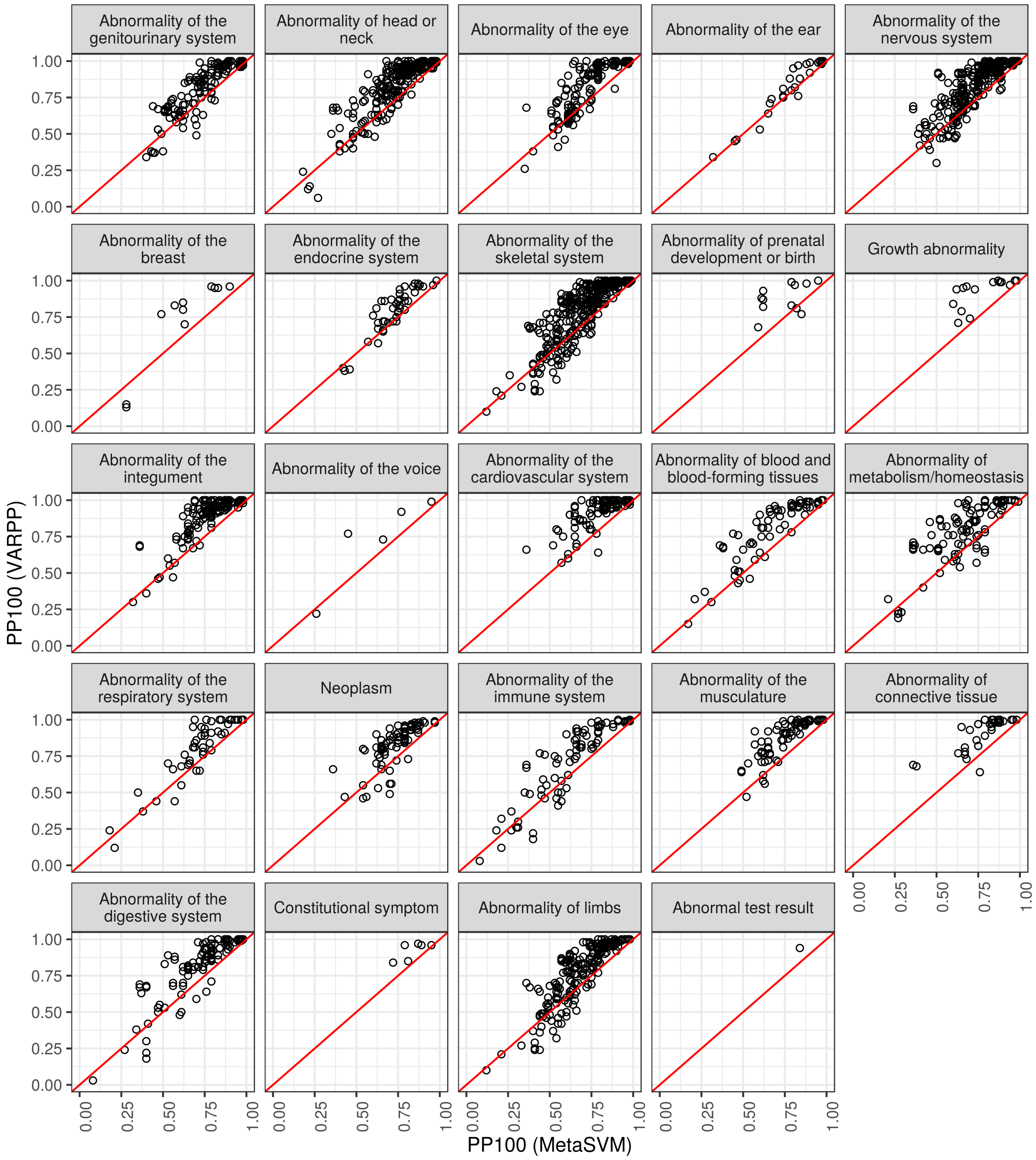

Supplementary Figure 8: Agreement scatter plots comparing the PP100 for VARPP including MetaSVM + GTEx specificity (*y* axis) versus the PP100 for MetaSVM scores alone (*x* axis) across broad HPO disease groups. The red line is the line of identity.

Variable importances for the top 30 improved HPO terms for VARPP classifiers including CADD and GTEx specificity

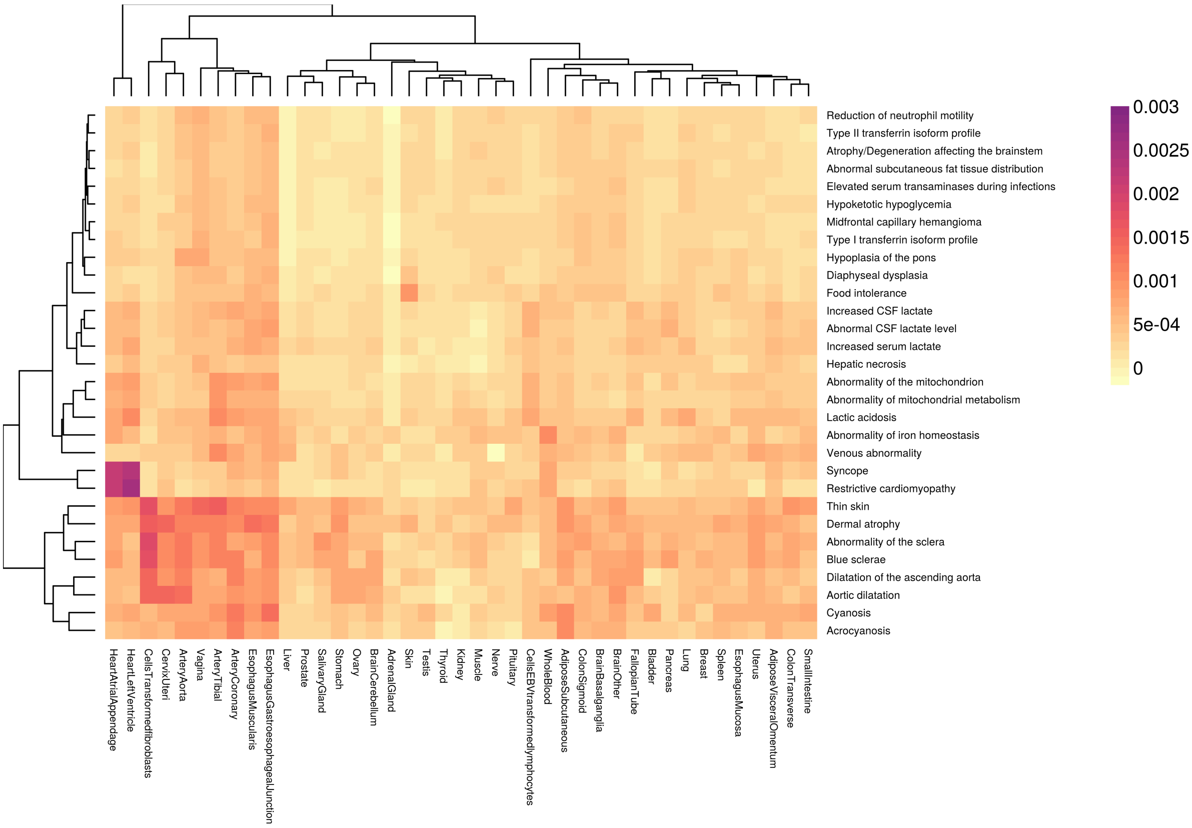

Supplementary Figure 9: Heatmap of GTEx tissue variable importances for the top 30 improved HPO terms (based on auPRC) for VARPP classifiers including CADD + GTEx specificity. The `aheatmap()` function from the R NMF package was used to plot the heatmap and we used the magma colour scale from the R viridis package. Rows and columns were clustered using the Euclidean distance measure and complete agglomeration.

Variable importances for the top 30 improved HPO terms for VARPP classifiers including MetaSVM and GTEx specificity

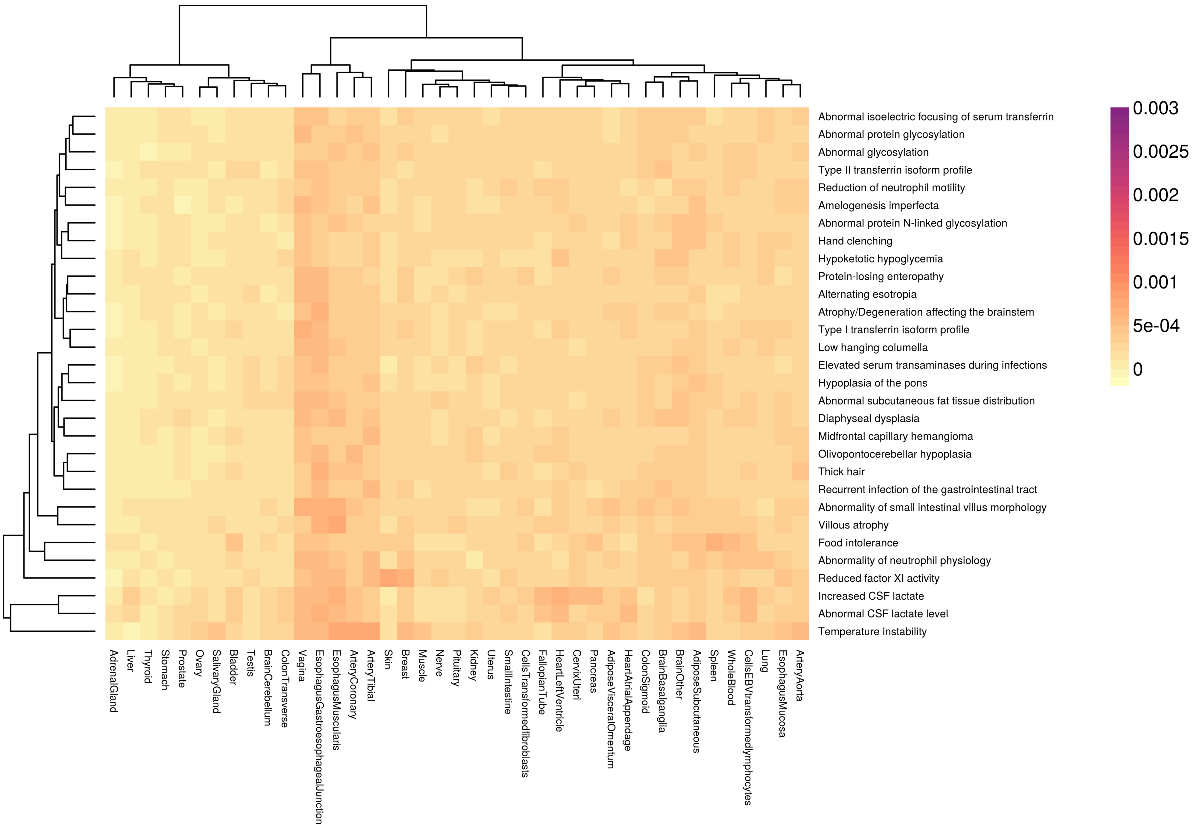

Supplementary Figure 10: Heatmap of GTEx tissue variable importances for the top 30 improved HPO terms (based on auPRC) for VARPP classifiers including MetaSVM + GTEx specificity. The `aheatmap()` function from the R NMF package was used to plot the heatmap and we used the magma colour scale from the R viridis package. Rows and columns were clustered using the Euclidean distance measure and complete agglomeration.

Variable importances for the top 30 improved HPO terms for VARPP classifiers including CADD and GTEx specificity

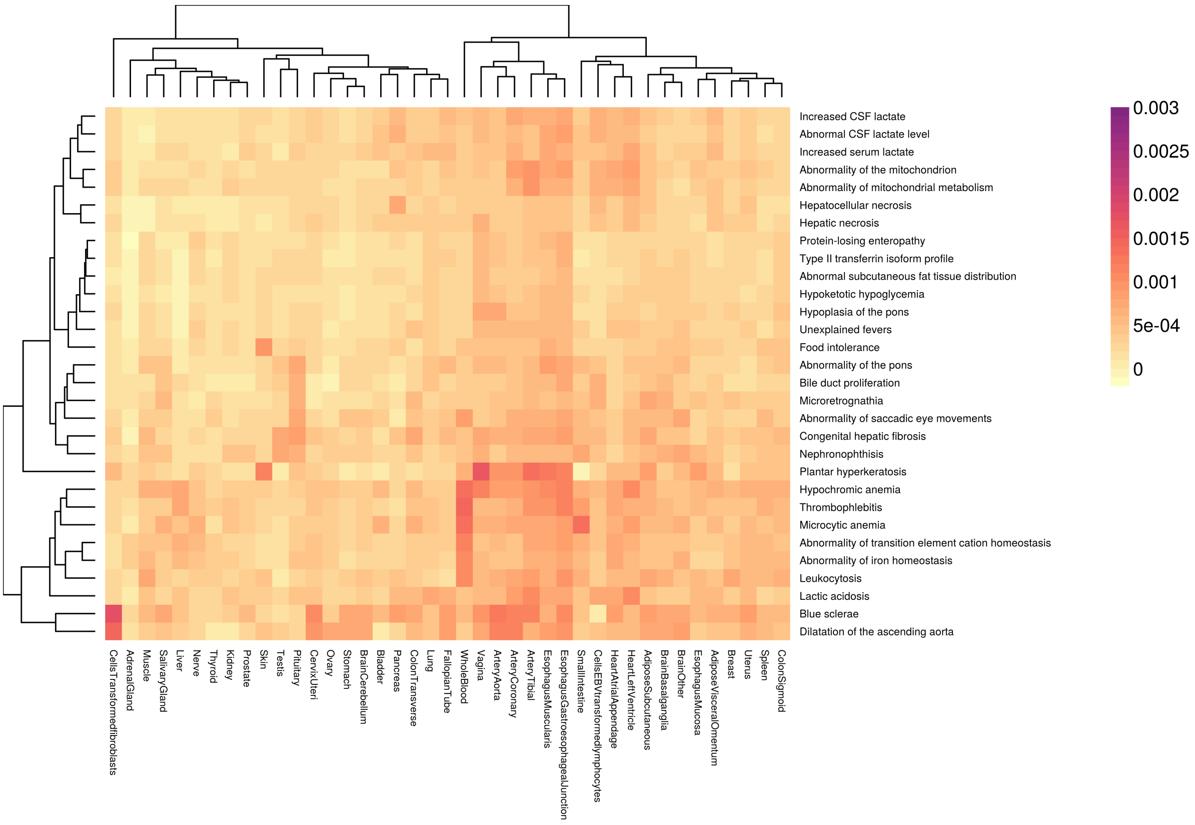

Supplementary Figure 11: Heatmap of GTEx tissue variable importances for the top 30 improved HPO terms (based on PP100) for VARPP classifiers including CADD and GTEx specificity. The `aheatmap()` function from the R NMF package was used to plot the heatmap and we used the magma colour scale from the R viridis package. Rows and columns were clustered using the Euclidean distance measure and complete agglomeration.

Variable importances for the top 30 improved HPO terms for VARPP classifiers including MetaSVM and GTEx specificity

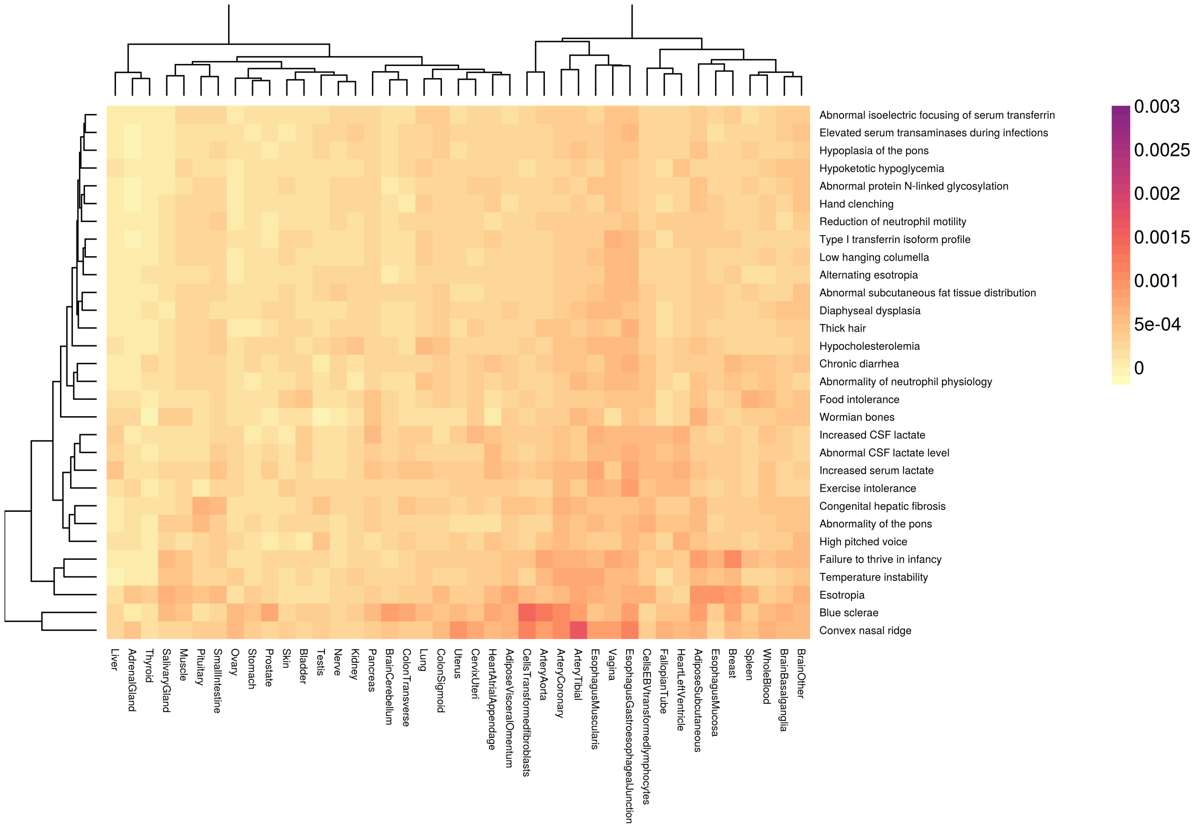

Supplementary Figure 12: Heatmap of GTEx tissue variable importances for the top 30 improved HPO terms (based on PP100) for VARPP classifiers including MetaSVM and GTEx specificity. The `aheatmap()` function from the R NMF package was used to plot the heatmap and we used the magma colour scale from the R viridis package. Rows and columns were clustered using the Euclidean distance measure and complete agglomeration.

VARPP performance in simulated disease exomes

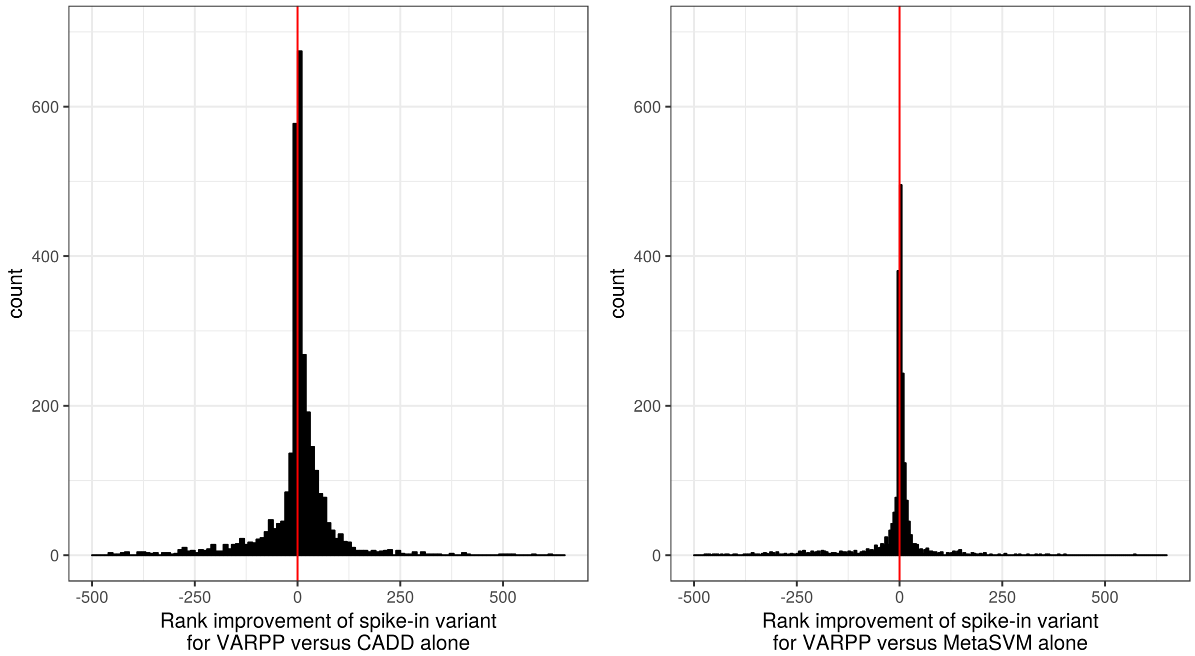

Supplementary Figure 13: Distribution of rank improvement for each spike-in variant. **Left** Rank improvement for VARPP classifiers including GTEx specificity + CADD versus use of CADD alone. **Right** Rank improvement for VARPP classifiers including GTEx specificity + MetaSVM versus use of MetaSVM alone. Bin width is equal to 10 and the vertical red line is equal to zero.

VARPP methodology overview

Gene 1	v1	v2	v3	v4			
Gene 2	v1	v2	v3	v4	v5	v6	v7
Gene 3	v1						
Gene 4	v1	v2	v3	v4	v5		
Gene 5	v1	v2	v3				
Gene 6	v1	v2	v3	v4	v5	v6	v7
Gene 7	v1	v2	v3	v4	v5	v6	v7
Gene 8	v1	v2	v3	v4	v5	v6	v7
Gene 9	v1	v2	v3	v4	v5	v6	v7
Gene 10	v1	v2	v3	v4	v5	v6	v7

Red = ClinVar pathogenic variants within genes associated with HPO phenotypic abnormality term(s)
Blue = Benign variants
* Benign variants are selected from genes that have no reported ClinVar variants, hence these genes do not overlap the genes containing ClinVar pathogenic variants.

Features associated with each variant are used by the classifier and include CADD or MetaSVM scores in conjunction with either cell or tissue-specific gene expression.

Sample genes with replacement, followed by selection of a single variant for in-bag sample (the same variant is selected for repeatedly sampled genes). Genes not selected will be the out-of-bag sample.

In-bag sample

Gene 1	v2
Gene 4	v5
Gene 4	v5
Gene 4	v5
Gene 4	v5
Gene 5	v1
Gene 6	v3
Gene 8	v7
Gene 9	v1
Gene 10	v3

Grow tree using in-bag sample

At each node ◯, a subset ($\sqrt{\# \text{ features}}$) of the features are randomly chosen and the best splitter is selected

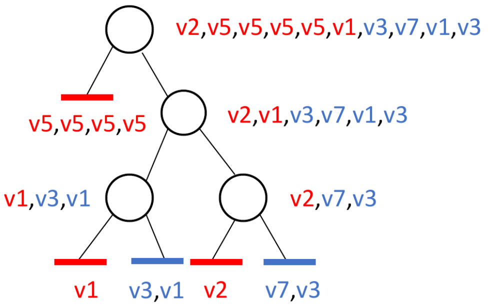

Pipe out-of-bag sample down tree

Tree casts a vote where 1=Pathogenic and 0=Benign

**Out-of-bag sample**								**Tree vote**						
Gene 2	v1	v2	v3	v4	v5	v6	v7	1	1	0	1	0	1	1
Gene 3	v1							1						
Gene 7	v1	v2	v3	v4	v5	v6	v7	0	0	1	0	0	1	0

Tree votes for out-of-bag samples are collected across all trees in the forest and the predicted probability of pathogenicity for each variant is calculated as the number of pathogenic predictions divided by the number of trees casting votes.

Supplementary Figure 14: Overview of VARPP methodology for disease-specific classifiers. VARPP uses random forests to predict pathogenicity of variants by disease, whilst accounting for clustering of variants within genes. This is achieved through a two-stage bootstrap approach which involves bootstrap sampling with replacement at the gene level, followed by selection of a single variant within each gene. This ensures that variants in the same gene can never be present in both in-bag and out-of-bag samples.

Top associated HPO term for each GTEx tissue

Supplementary Table 1: For each GTEx tissue (Tissue) the HPO term with the highest variable importance for that tissue is shown for VARPP including CADD + GTEx specificity (VARPP including CADD), and for VARPP including MetaSVM + GTEx specificity (VARPP including MetaSVM).

Tissue	VARPP including CADD	VARPP including MetaSVM
Adipose (subcutaneous)	Abnormal joint morphology	Abnormality of the nose
Adipose (visceral omentum)	Abnormality of the skeletal system	Abnormality of skeletal morphology
Adrenal gland	Muscular hypotonia	Abnormal muscle tone
Artery (aorta)	Abnormality of the mouth	Abnormality of the nervous system
Artery (coronary)	Abnormality of the skull	Abnormality of the nervous system
Artery (tibial)	Abnormal axial skeleton morphology	Abnormality of the nervous system
Bladder	Abnormality of the orbital region	Morphological abnormality of the central nervous system
Brain (basal ganglia)	Abnormality of movement	Abnormality of central motor function
Brain (cerebellum)	Abnormality of the integument	Abnormality of the integument
Brain (other)	Neurological speech impairment	Abnormality of central motor function
Breast	Abnormality of the kidney	Abnormality of the skeletal system
Cells (EBV transformed lymphocytes)	Abnormality of the female genitalia	Abnormality of the face
Cells (transformed fibroblasts)	Abnormality of the skull	Abnormality of the skull
Cervix uteri	Aplasia/hypoplasia involving the skeleton	Abnormality of nervous system physiology
Colon (sigmoid)	Abnormality of the nasal bridge	Abnormality of nervous system morphology
Colon (transverse)	Abnormality of the nose	Abnormality of nervous system morphology
Oesophagus (gastroesophageal junction)	Abnormality of the nervous system	Abnormality of the nervous system

Oesophagus (mucosa)	Abnormality of the dentition	Generalised abnormality of skin
Oesophagus (muscularis)	Abnormal eye morphology	Abnormality of the nervous system
Fallopian tube	Abnormality of the nervous system	Abnormality of nervous system physiology
Heart (atrial appendage)	Abnormality of the musculature	Abnormality of the musculature
Heart (left ventricle)	Abnormality of muscle physiology	Abnormality of the head
Kidney	Abnormal renal physiology	Abnormality of metabolism/homeostasis
Liver	Abnormality of metabolism/homeostasis	Abnormality of metabolism/homeostasis
Lung	Abnormality of the nervous system	Abnormality of the face
Muscle	Abnormality of the musculature	Abnormality of the musculature
Nerve	Abnormality of the curvature of the vertebral column	Abnormality of the skeletal system
Ovary	Abnormality of the outer ear	Abnormality of nervous system physiology
Pancreas	Abnormality of the nervous system	Global developmental delay
Pituitary	Abnormality of nervous system morphology	Abnormality of nervous system morphology
Prostate	Abnormality of central motor function	Abnormality of central motor function
Salivary gland	Abnormality of the oral cavity	Abnormality of the globe
Skin	Abnormality of skin adnexa morphology	Abnormality of skin adnexa morphology
Small intestine	Generalised abnormality of skin	Abnormality of the nervous system
Spleen	Abnormality of bone marrow cell morphology	Abnormality of the nervous system
Stomach	Intellectual disability	Abnormality of the nervous system

Testis	Abnormality of metabolism/homeostasis	Abnormality of metabolism/homeostasis
Thyroid	Abnormality of oral cavity	Abnormality of the face
Uterus	Abnormality of nervous system physiology	Abnormality of nervous system physiology
Vagina	Abnormality of skin adnexa morphology	Abnormality of skin adnexa morphology
Whole blood	Abnormality of blood and blood-forming tissues	Abnormality of blood and blood-forming tissues
