# Supplementary material for: Personalised analytics for rare disease diagnostics
Source: Nat Commun. 2019 Nov 21;10:5274. doi: 10.1038/s41467-019-13345-5 (PMC6872807; doi:10.1038/s41467-019-13345-5)
Supplement: Supplementary file 3 — Description of additional Supplementary Files [file 41467_2019_13345_MOESM3_ESM.pdf]

Description of Additional Supplementary Files

File Name: Supplementary Data 1
Description: **Performance results for VARPP**
This file contains performance results (auPRC and PP100), across all 1,879 HPO Phenotypic Abnormality terms, for the VARPP classifiers described in the main publication.

File Name: Supplementary Data 2
Description: **Variable importance measures for the 1,879 HPO terms described in the main publication**
This file contains variable importances, across all 1,879 HPO Phenotypic Abnormality terms, for the VARPP classifiers described in the main publication.

File Name: Supplementary Data 3
Description: **Performance results for VARPP for simulated disease exomes**
This file contains performance results for the simulated disease exomes described in the main publication.

File Name: Supplementary Data 4
Description: **Pathogenic variants for the 1,879 HPO terms described in the main publication**
This file contains a R list of ClinVar pathogenic variants for the 1,879 HPO terms considered in our study (as described in Methods: Pathogenic variants). The seed genes associated with each HPO term were determined using Phenolyzer. ClinVar pathogenic variants within the seed genes were selected using annotation from dbNSFP. Annotation in this file includes the gene symbol (Gene), MetaSVM scores (MetaSVM_rankscore), CADD scores (CADD_raw_rankscore), pathogenic indicator (Pathogenic) and an identifier for each gene/variant combination (GeneVariant). The file can be loaded using the R `load()` function.

File Name: Supplementary Data 5
Description: **Benign variants**
This file contains the benign variants used in our study with selected dbNSFP annotations (as described in Methods: Benign variants). Benign variants were defined as those not present in ClinVar that are also common in at least one large sequencing cohort. Annotation in this file includes:
X.chr: Chromosome number
pos.1.based.: Physical position on the chromosome as to hg38
ref: Reference nucleotide allele (as on the + strand)
alt: Alternative nucleotide allele (as on the + strand)
aaref: Reference amino acid
aaalt: Alternative amino acid
rs_dbSNP147: rs number from dbSNP 147
hg19_chr: Chromosome as to hg19
hg19_pos.1.based.: Physical position on the chromosome as to hg19
hg18_chr: Chromosome as to hg18
hg18_pos.1.based.: Physical position on the chromosome as to hg18
Gene: Gene symbol
MetaSVM_rankscore: MetaSVM scores
CADD_raw_rankscore: CADD scores
X1000Gp3_AF: Alternative allele frequency in the whole 1000 genomes phase 3 data
TWINSUK_AF: Alternative allele frequency in called genotypes in UK10K TWINSUK cohort
ALSPAC_AF: Alternative allele frequency in called genotypes in UK10K ALSPAC cohort

ESP6500_AA_AF: Alternative allele frequency in the African American samples of the NHLBI GO Exome Sequencing Project
ESP6500_EA_AF: Alternative allele frequency in the European American samples of the NHLBI GO Exome Sequencing Project
ExAC_AF: Allele frequency in total ExAC samples
variant: Variant identifier within each gene
GeneVariant: Variant identifier for each gene/variant combination

File Name: Supplementary Data 6
Description: **GTEx expression data**
This file contains the GTEx gene expression data used in our study (as described in Methods: Gene expression data). In addition to gene expression data across the GTEx tissues, the file also contains the Ensembl gene identifier (ensemble_gene_id), gene symbol (hgnc_symbol), chromosome (chromosome_name), genome start position (start_position), genome end position (end_position), gene biotype (gene_biotype) and an indicator as to whether the gene contain ClinVar pathogenic variants (Pathogenic).

File Name: Supplementary Data 7
Description: **FANTOM5 cell groups used for normalisation**
This file contains the FANTOM5 cell groups used for normalisation and averaging of gene expression (as described in Methods: Gene expression data).

File Name: Supplementary Data 8
Description: **FANTOM5 expression data**
This file contains the FANTOM5 gene expression data used in our study (as described in Methods: Gene expression data). In addition to gene expression data across the FANTOM5 cell types, the file also contains the gene identifier (hgnc_id), gene symbol (hgnc_symbol), gene biotype (locus_group & locus_type), chromosomal location (location), chromosome (chromosome) and an indicator as to whether the gene contain ClinVar pathogenic variants (Pathogenic).

File Name: Supplementary Data 9
Description: **GTEx specificity data**
This file contains the GTEx gene specificity data used in our study (as described in Methods: Specificity of gene expression). In addition to gene specificity data across the GTEx tissues, the file also contains the Ensembl gene identifier (ensemble_gene_id), gene symbol (hgnc_symbol), chromosome (chromosome_name), genome start position (start_position), genome end position (end_position), gene biotype (gene_biotype) and an indicator as to whether the gene contain ClinVar pathogenic variants (Pathogenic).

File Name: Supplementary Data 10
Description: **FANTOM5 specificity data**
This file contains the FANTOM5 gene specificity data used in our study (as described in Methods: Specificity of gene expression). In addition to gene specificity data across the FANTOM5 cell types, the file also contains the gene identifier (hgnc_id), gene symbol (hgnc_symbol), gene biotype (locus_group & locus_type), chromosomal location (location), chromosome (chromosome) and an indicator as to whether the gene contain ClinVar pathogenic variants (Pathogenic).

File Name: Supplementary Data 11
Description: **ClinVar pathogenic spike-in variants**
ClinVar pathogenic variants that were spiked into 1000 Genomes samples. Annotation in this file includes:

X.chr: Chromosome number
pos.1.coor.: Physical position on the chromosome as to hg19
ref: Reference nucleotide allele (as on the + strand)
alt: Alternative nucleotide allele (as on the + strand)
aaref: Reference amino acid
aaalt: Alternative amino acid
genename: Gene symbol
MetaSVM_rankscore: MetaSVM scores
CADD_raw_rankscore: CADD scores
X1000Gp1_AF: Alternative allele frequency in the whole 1000 genomes phase 1 data
X1000Gp1_AFR_AF: Alternative allele frequency in the 1000 genomes phase 1 African descendent samples
X1000Gp1_EUR_AF: Alternative allele frequency in the 1000 genomes phase 1 data European descendant samples
X1000Gp1_AMR_AF: Alternative allele frequency in the 1000 genomes phase 1 data American descendant samples
X1000Gp1_ASN_AF: Alternative allele frequency in the 1000 genomes phase 1 data Asian descendant samples
ESP6500_AA_AF: Alternative allele frequency in the African American samples of the NHLBI GO Exome Sequencing Project
ESP6500_EA_AF: Alternative allele frequency in the European American samples of the NHLBI GO Exome Sequencing Project
ARIC5606_AA_AF: Alternative allele frequency of 2403 exomes of African Americans from the Atherosclerosis Risk in Communities Study
ARIC5606_EA_AF: Alternative allele frequency of 3203 exomes of European Americans from the Atherosclerosis Risk in Communities Study
ExAC_AF: Allele frequency in total ExAC samples
clinvar_clnsig: ClinVar clinical significance
clinvar_trait: ClinVar trait/disease
clinvar_golden_stars: ClinVar review status summary
ClinVar_id: ClinVar identifier for variant
Name: ClinVar description of variant effect
HPO: HPO term(s) associated with ClinVar variant

File Name: Supplementary Data 12
Description: **Simulated disease exomes**
This zip file contains the three spike-in batches described in Methods (Simulated disease exomes). Each batch contains the 1000 genomes data with a single spiked-in pathogenic variant (`spike_dbNSFP`), data required to run VARPP (`vp_spike, dbNSFP, gtex_specificity_percentile, spike_batch#`) and the custom function used for the analysis (`TwoStageBootSpikeRF()`). Each batch can be loaded in R using the `load()` function. Each batch was run on a cluster using the following commands:

```
library(parallel)
spike_predict <- list()
cl <- makeCluster(13, outfile=”log.txt”)
clusterExport(cl, list(“spike_batch#”, “TwoStageBootSpikeRF”,
“vp_spike”, “dbNSFP”, “gtex_specificity_percentile”,
“spike_dbNSFP”))
clusterEvalQ(cl, library(ranger))
clusterEvalQ(cl, library(dplyr))
clusterEvalQ(cl, library(caret))
```

```
spike_predict$GTEx_specificity <- parSapply(cl, names(spike_dbNSFP),
FUN=function(x) TwoStageBootSpikeRF(ntree=2000,
expr=gtex_specificity_percentile, hpoterms=tail(spike_dbNSFP[[x]],
1)[ , “HPO”], patient=spike_dbNSFP[[x]], batch=spike_batch#),
simplify=FALSE)
stopCluster(cl)
```
